# Molecular characterisation of human adenoviruses associated with respiratory infections in Uganda

**DOI:** 10.1186/s12879-023-08403-9

**Published:** 2023-06-27

**Authors:** Qouilazoni A. Ukuli, Bernard Erima, Andrew Mubiru, Gladys Atim, Titus Tugume, Hannah Kibuuka, Edison Mworozi, Mariette F. Ducatez, Fred Wabwire-Mangen, Denis K. Byarugaba

**Affiliations:** 1 Makerere University Walter Reed Project, Kampala, Uganda; 2 College of Health Sciences, Makerere University, P.O. Box 7062, Kampala, Uganda; 3 IHAP, UMR1225, Université de Toulouse, INRAE, ENVT, Toulouse, France; 4 School of Public Health, Makerere University, P.O. Box 7062, Kampala, Uganda; 5 College of Veterinary Medicine, Makerere University, P.O. Box 7062, Kampala, Uganda

**Keywords:** Human adenoviruses, Respiratory infection, Uganda

## Abstract

Human adenoviruses (HAdV) are a diverse group of viruses causing a broad range of infections of the respiratory, urogenital and gastrointestinal tracts and keratoconjunctivitis. There are seven species of human adenoviruses with 113 genotypes which may contain multiple genetic variants. This study characterised respiratory human adenoviruses and associated factors in samples collected from selected hospitals in Uganda. A total of 2,298 nasopharyngeal samples were collected between the period of 2008 to 2016 from patients seeking health care at tertiary hospitals for influenza-like illness. They were screened by polymerase chain reaction (PCR) to determine the prevalence of HAdV. HAdV was cultured in A549 cell lines and the hexon gene was sequenced for genotyping. Of the 2,298 samples tested, 225 (9.8%) were adenovirus-positive by PCR. Age was found to be significantly associated with HAdV infections (*p* = *0.028*) with 98% (220/225) of the positives in children aged 5 years and below and none in adults above 25 years of age. The sequenced isolates belonged to species HAdV-B and HAdV-C with most isolates identified as genotype B3. The results showed a high prevalence and genetic diversity in respiratory HAdV circulating in Ugandan population. Deeper genomic characterization based on whole genome sequencing may be necessary to further elucidate possible transmission and impact of current adenovirus-vectored vaccines in Africa.

## Background

Globally, human adenoviruses (HAdV) have been implicated in a broad spectrum of both sporadic and epidemic disease outbreaks [1, 2]. Since their first isolation in 1953 [3], human adenoviruses have evolved into a diverse group of viruses causing a broad range of infections with significant human morbidity, mortality and world-wide spread [4, 5]. They cause infections of the lower and upper respiratory tract illnesses, keratoconjunctivitis, urinogenital infections and acute gastroenteritis in both children and adults [2, 6]. Although adenovirus infections are generally self-limiting [7], human adenoviruses types HAdV-B3, HAdV-B7 and HAdV-E4 have been implicated in both upper and lower respiratory tract infection sometimes with fatal outcomes in both immunocompromised and immune-competent individuals [7, 8].

There are a number of adenovirus pathogens circulating within populations. Up to 113 HAdV genotypes have been recently characterized using whole genome analysis and these include the 51 serotypes originally recorded [9]. These have been classified into seven species under the genus *Mastadenoviruses* [10]. The seven species; HAdV-A to HAdV-G have been classified based on their biological properties and DNA homology [11]. Characterisation was initially done using serologyand sequencing of the hexonand fiber protein genes but has evolved to include the penton base protein gene and recently; whole genome sequencing (WGS) [9, 12]. Whole genome sequencing provides a clear discrimination of genotypes including recombinants [13]. Species HAdV-B (types 3, 4, 7, 11, 14, 16, 21, 34, 35 and 55), HAdV-C (types 1, 2, 5 and 6) and HAdV-E (type 4) are not only associated with respiratory tract infections but may also be involved in other infections in humans [2, 14].

As the threat for novel and re-emerging infections with pandemic capability increases, there is a justifiable need to carry out baseline surveillance and genetic analysis of endemic pathogens circulating within populations with WGS provifing for the availability of high-resolution genomic data facilitating a deeper insight into the molecular evolution of human adenoviruses [15].

The emergence of novel adenovirus strains has been attributed to recombination of genomes of different viral strains and mutations serving as a major pathway in the molecular evolution of new types and emergent pathogens [7, 16]. Recombination may lead to altered pathogenicity and altered tissue specificities [2] such as HAdV-B55, a highly contagious HAdV type which is a recombinant of HAdV-B11 hexon gene and HAdV-B14 penton base and fiber protein genes with altered tissue tropism and antibody specificity [9, 13].

Human adenovirus is one of the many respiratory disease pathogens that present with influenza like symptoms. In Uganda, influenza like illnesses (ILI) are common with 46.6% of samples from those ILI cases yielding one or more respiratory viruses of which 8.7% are adenoviruses [17]. However, adenovirus outbreaks are not usually reported as they most likely go undetected due to lack of awareness of adenoviruses as causative agents and scarcity of diagnostic tests. Identification and characterization of new and re-emerging adenoviruses is important in the prevention and control of disease outbreaks as it would aid in predicting and preparing for future disease occurrences. As more and more adenovirus-vectored vaccines get approval for use against pandemic diseases, knowledge of circulating adenoviruses is critical to inform deployment of such vaccines in those locations following increased risk in adenovirus seropositive populations.

## Material and methods

A retrospective analysis was carried out on samples collected between the period of 2008 and 2016 from patients with influenza-like illness at selected health care facilities. These samples were collected from established sentinel sites for Makerere University Walter Reed Project Influenza surveillance programme: Mulago national neferral hospital in Kampala city, Kayunga district hospital, Bugiri district hospital, Gulu regional referral hospital and Jinja Regional Referral Hospital. A total of 2,298 nasopharyngeal swab samples were collected from individuals aged 6 months and above presenting at the hospitals’ outpatient department with influenza-like symptoms; a fever (above 38^0^C) plus either cough or sore throat within 72 h of presentation. Samples were collected using a dacron swab in a 2-ml cryovial containing virus transport medium enriched with antibiotics and stored at a central laboratory facility at -80 °C until processing.

### Detection and isolation of adenoviruses

In order to detect presence of adenoviruses, total nucleic acid (TNA) was extracted from samples of nasopharyngeal swabs using Qiagen QIAamp mini elute virus spin extraction kit (Qiagen, Germany), Ref: 57,704 according to manufacturer’s instructions.

Polymerase chain reaction (PCR) targeting the hexon gene was performed using primers; Adv-Forward: GCC CCA GTG GTC TTA CAT GCA CAT C andAdv- reverse: GCC ACG GTG GGG TTT CTA AAC TT.

A 25 µl reaction mix composed of: 2.5 µl of 10 × PCR buffer, 0.75 µl of 50 mM MgCl_2,_ 0.5 µl of 10 mM dNTP mix, 1 µl 10 µM of each of the reverse and forward primers, 0.1 µl of platinum taq polymerase (Invitrogen, USA), 18.15 µl PCR molecular grade water and 1 µl of DNA extract as template. The PCR conditions were as follows: Initial denaturation at 94 °C for 10 min, followed by 40 cycles of 94ºC for 30 s, 52ºC for 30 s and 72ºC for 1 min, and final extension at 72ºC for 10 min. The PCR product was run on a 2% agarose gel with an expected band product of 301 bp sized against a DNA molecular ladder.

Adenovirus PCR-positive samples (100 µl) were inoculated on 90% confluent A549 cell line in T25 culture flasks pre-washed with trypsin-DPBS (Trypsin Dulbecco’s Phosphate Buffered Saline) to facilitate virus entry. The flasks were incubated in a tissue culture incubator at 37 °C with 5% CO_2_ and followed up for 7 days.

For genotyping, PCR targeting a region in the conserved loop 4 of the hexon gene was done according to previously described methods by Sriwanna et al.[18] using primers:

- Adv-F2: TTY CCC ATG GCN CAC AAC AC and
- Adv- 2 V: GYY TCR ATG AYG CCG CGG TG.

A 25 µl reaction mix composed of: 2.5 µl of 10 × PCR buffer, 0.75 µl of 50 mM MgCl_2,_ 0.5 µl of 10 mM dNTP mix, 1 µl 10 µM of each of the reverse and forward primers, 0.1 µl of platinum taq polymerase, 18.15 µl PCR molecular grade water and 1 µl of DNA extract as template. The PCR conditions were as follows: Initial denaturation at 94 °C for 3 min followed by 40 cycles of 94 °C for 30 s, 50 °C for 30 s, and 72 °C for 1 min and 45 s with a final extension at 72 °C for 10 min.

Amplicons from PCR were run on a 2% agarose gel in electrophoresis using a UV light gel trans-illuminator after staining with gel red. DNA products were extracted from the gel matrix and purified using the Qiagen QIAamp gel extraction kit (Qiagen, Germany) ref: 28,704 according to manufacturer’s instructions.

### Sequencing and phylogenetic analysis

Purified amplicons of partial hexon gene fragment were subjected to nucleotide sequencing by Sanger sequencing method using the BigDye Terminator v3.1, Cycle Sequencing Kit (Thermo Fisher Scientific Inc., U.S.A) according to the manufacturer’s instructions. Sequencher software Version 5.0 (Gene Codes Corporation, USA) was used to edit and assemble the raw sequence data. The sequences were aligned against reference human adenovirus hexon gene sequences from GenBank using Muscle offered in MEGA X software. The BLASTn program by National Center for Biotechnology Information, Bethesda, MD, USA was used to identify homologous nucleotide sequences in the GenBank database and to identify the genotypes of the isolates. The most related sequences based on their E-score from Genbank were used in the analysis. Phylogenetic analysis and construction of phylogenetic trees was done using the neighbor-joining and Maximum-composite-likelihood methods offered in MEGA X. The reference sequences from GenBank used in the analysis were: HQ003817, JX423389, AB601020, AJ293901, EF486502, X74662, AY737797, AY737798, NC_003266, AB448767, EF121005, NC_001460, DQ149611, GU191019, AB330121, HM565136. The robustness of the phylogenetic trees was statistically evaluated by bootstrap analysis with 1000 replicates. The nucleotide sequences were compared phylogenetically using the General time reversible model with a Maximum-composite-likelihood and Neighbour joining method to infer the evolutionary history. The phylogenetic trees were edited using Fig tree version 1.4.4.

## Results

### Prevalence of human respiratory adenovirus infections and associated factors

The observed prevalence of human respiratory adenovirus infections in this study was 9.8% (225/2298) in nasopharyngeal swab samples collected from selected hospitals from Uganda. A higher prevalence (10.3% *n* = 219) was observed among children aged between 0.5 and 5 years (Table 1) compared to older children and adults. In the different hospitals, there was an uneven distribution of HadV infections with Gulu Regional Referral Hospital showing the highest number of cases (15.4%, *n* = 227) followed by Mulago National Referral Hospital (11.7%, *n* = 1107) (Table 1). Children aged between 0.5 and 5 years were more likely to acquire HAdV infections than higher age groups (*p* = *0.028*) (Table 1).

**Table 1:** Association between HadV and the demographic characteristics; Age and site were significantly associated with HAdV infection (*p* = *0.028* and *p* = *0* respectively). More males (10.1%) than females (9.5%) tested positive for the virus. Children aged between 0.5 and 5 years had the highest proportion of positives (10.3%) while there were no positives detected in samples for participants above 25 years and above

Adenovirus *N* = 2298				
**Characteristics**		**Number of samples tested (n)**	**Positive (%) *n* = 225**	***p*-value**
**Site**	Bugiri	99	5.1	0
	Gulu	227	15.4	
	Jinja	837	6.70	
	Mulago	1107	11.7	
	Kayunga	28	0.0	
**Sex**	Male	1197	10.1	0.603
	Female	1101	9.5	
**Age**	0.5–5 years	2137	10.3	0.028
	6–15 years	106	3.8	
	16–25 years	30	3.3	
	More than 25 years	25	0.0	

### Respiratory HAdV infections in different hospitals over the years

**Fig. 1:**
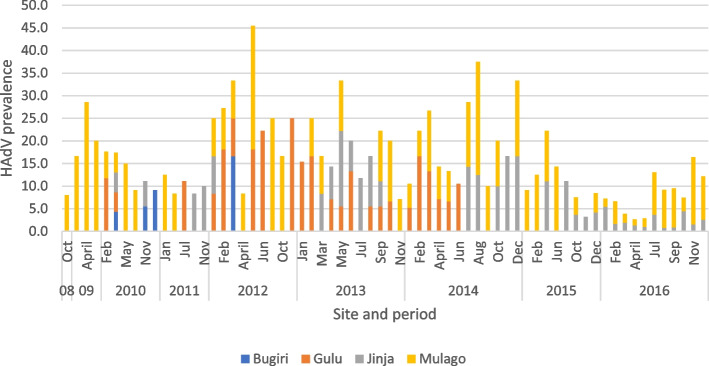
Distribution of HAdV within different Hospitals over the years; The Highest HAdV infections were observed in 2012 and 2014 for Gulu Regional Referral Hospital and Mulago National Referral Hospital and 2013 for Jinja Regional Referral Hospital

**Fig. 2:**
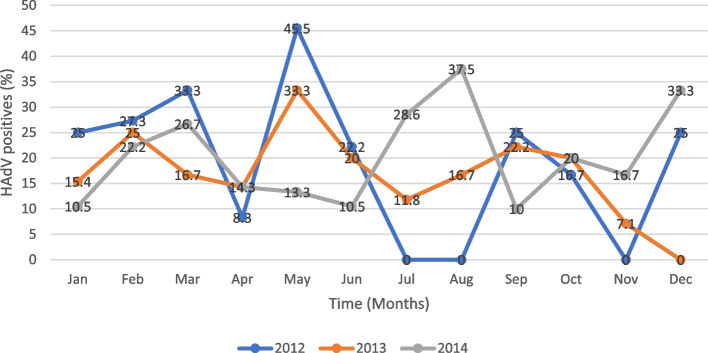
Distribution of HAdV per month for the years 2012, 2013 and 2014; A higher prevalence (45.5% and 33.3%) was observed in the month of May of 2012 and 2013 respectively but lowest (0%) in months of July, August and November for the year 2012. For the year 2014, the highest prevalence was observed in the month of August (37.5%), followed by December (33.3%) and lastly July (28.6%). The disease occurrence generally displays an oscillating pattern over the months

Human adenovirus infections over the years were observed to gradually decrease across the hospital sites with the highest peaks being obtained between 2012 to 2014 for Mulago National Referral Hospital and Gulu Regional Referral Hospital (Fig. 1). There was gradual increase of infections over the years with the highest peak being attained in 2012 after which the positivity started dropping to lower figures. The year of 2012 had the highest HAdV prevalence of 19.5% and 2008 had the lowest prevalence of 3.8% (Fig. 2).

Line graphs of time (months) against prevalence for the years 2012, 2013 and 2014 with the highest prevalence peaks are showed below (Fig. 2).

### Genetic diversity

Analysis of nucleotide diversity in the aligned sequences of the HAdV isolates showed variation in their nucleotides. Sequences of the same HAdV species displayed similar nucleotide variations. The genetic distances analysis showed close similarity of sequences within the same species and a distinct variation between different species (Table 2).

**Table 2:** Genetic distances between the Isolates; The number of base substitutions per site between sequences are shown. Analyses of the number of substitutions per site were conducted using the Tamura-Nei model. Codon positions included were 1st + 2nd + 3rd + Noncoding. The mean genetic distance was 0.16. All ambiguous positions were removed for each sequence pair (pairwise deletion option)

ISOLATE ID	C1	C2	C5	C6	C57	B3	B7	B11	D8
2,009,100,062 MUWRP/AdV/0002/2016	0.239	0.259	0.229	0.269	0.248	0.002	0.017	0.148	0.201
2,009,100,873 MUWRP/AdV/0006/2016	0.285	0.293	0.270	0.301	0.323	0.004	0.018	0.149	0.238
2,012,100,252 MUWRP/AdV/0021/2016	0.249	0.288	0.241	0.290	0.268	0.012	0.018	0.155	0.214
2,013,100,155 MUWRP/AdV/0025/2016	0.284	0.285	0.284	0.294	0.327	0.000	0.015	0.155	0.212
2,013,100,169 MUWRP/AdV/0027/2016	0.093	0.129	0.006	0.134	0.098	0.267	0.267	0.293	0.229
2,013,100,451 MUWRP/AdV/0032/2016	0.266	0.294	0.263	0.293	0.270	0.007	0.022	0.164	0.228
2,013,101,296 MUWRP/AdV/0038/2016	0.239	0.258	0.229	0.268	0.248	0.002	0.017	0.147	0.201
2,014,100,272 MUWRP/AdV/0041/2016	0.261	0.297	0.253	0.302	0.280	0.003	0.009	0.162	0.217
2,014,100,445 MUWRP/AdV/0046/2016	0.132	0.033	0.148	0.055	0.122	0.289	0.302	0.310	0.252
2,014,100,818 MUWRP/AdV/0049/2016	0.286	0.315	0.281	0.322	0.317	0.025	0.018	0.142	0.208
2,014,101,117 MUWRP/AdV/0054/2017	0.020	0.138	0.110	0.143	0.041	0.258	0.259	0.260	0.227
2,016,100,556 MUWRP/AdV/0063/2017	0.088	0.121	0.000	0.129	0.092	0.253	0.257	0.281	0.218
2,016,100,594 MUWRP/AdV/0065/2017	0.163	0.050	0.175	0.025	0.156	0.306	0.328	0.319	0.258
2,016,100,625 MUWRP/AdV/0066/2017	0.274	0.295	0.263	0.302	0.305	0.019	0.027	0.163	0.242
2,016,100,997 MUWRP/AdV/0078/2018	0.293	0.317	0.281	0.324	0.327	0.027	0.019	0.149	0.223

### Genotype characterisation

**Fig. 3:**
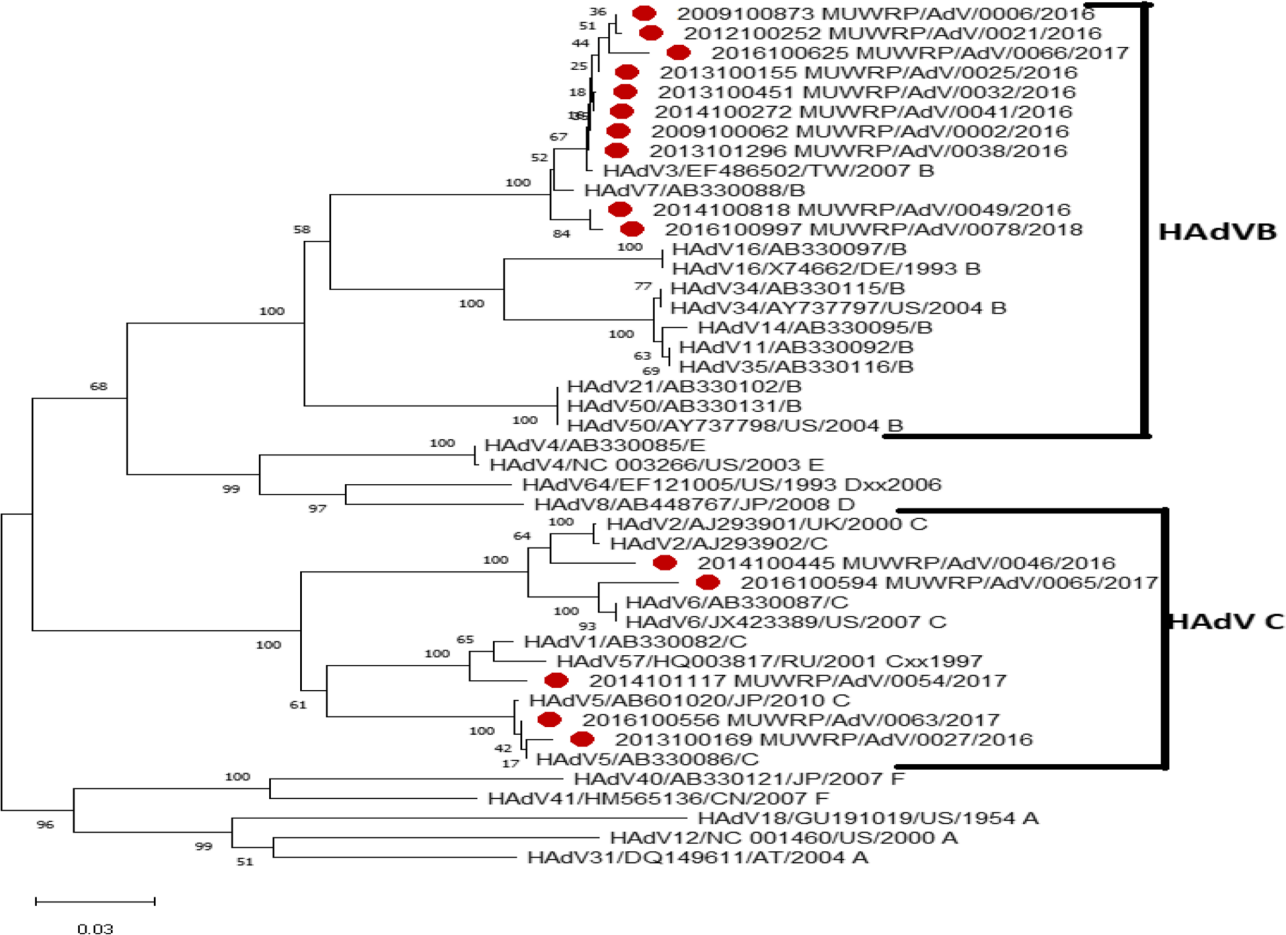
Phylogenetic tree of partial hexon gene of Ugandan HAdV isolates; The evolutionary history was inferred using the Neighbor-Joining method. The percentage of replicate trees in which the associated taxa clustered together in the bootstrap test (1000 replicates) are shown next to the branches. The evolutionary distances were computed using the Tamura-Nei method and are in the units of the number of base substitutions per site

Phylogenetic analysis identified 5 isolates of HAdV-C and 10 isolates of HAdV-B (Fig. 3). The difference in species distribution by site was small; however, more of HAdV B were seen in Mulago National Referral Hospital (MNRH). Genetic analysis identified genotypes: HAdV-C1, HAdV-C2, HAdV-C5, HAdV-C6, HAdV-B3 and HAdV-B7.

## Discussion

This study was carried out to determine the prevalence and genetic relationships of human adenoviruses associated with respiratory infections. Epidemiological investigations have revealed that multiple genetic variants may exist within a single particular genotype of HAdV [19] thus the need for routine surveillance and in-depth molecular characterization to identify the prevalence of the different adenovirus genetic variants. Adenovirus infection spread is usually dependant on age of an individual, geographic and seasonal difference dynamics and other demographic factors that affect disease occurrence such as close living conditions and the immune status of an individual [11, 20]. Studies have reported a varied prevalence of up to 49.6% in a military population in Korea [21], 8.7% in Uganda [17], 2.0– 6.1% in hospitalized patients in Guangzhou City, China [22] and among organ transplant patients, 64% were reported to have developed adenovirus disease [23].

A prevalence of 9.8% (*n* = 225) was observed in this study. This is in agreement with the 8.7% HAdV prevalence that was previously reported for Kampala and Entebbe by Balinandi et al. [17] and 9.4% in Uganda by Mimbe et al.[24]. The observed HAdV prevalence highlights the importance of HAdV detection in clinical settings and surveillance in communities. Indeed studies elsewhere in Africa have reported higher prevalence of up to 30.8% among 6381 patients in Senegal [25] and 19.6% in Tunisia [11].

It was observed that children below 5 years of age were more likely to be infected with HAdV than adults older than 15 years (*p* = *0.028*). Generally, children aged 5 years and below are more susceptible to respiratory viral infections as documented in various studies in sub-Saharan Africa [26]. A higher infection prevalence of 10.3% was observed among children less than 6 years. This is consistent with a study in China that reported HAdV detection rate of 8.55% and 9.4% in Chongqing and Hunan Province respectively in hospitalized children with Respiratory Tract Infections (RTIs) [3]. High infection rates of up to 19.5% have been recorded in Tunisia [26] among children with community acquired lower acute respiratory infections (LARIs) conforming to the trend of infection in our study. This could be attributed to the health-seeking behaviors of parents of infants which is more frequent than in adults and older children [27] as well as previous exposure during early childhood; most of the human population (90%) have been exposed to at least one serotype of HAdV [28].

Throughout the world, HAdV has been shown to cause between 5 and 15% of upper acute respiratory infections (UARIs) and about 5% of LARIs in children below 5 years [11]. This observation is routine for adenoviruses as seen in most of the published data that indicates children to be more susceptible to respiratory adenovirus infections [8, 29]. This is attributed to the immunity not being well developed in children thus making them more susceptible to various infections [2]. Furthermore, transmission among children easily occurs since they usually spend time playing together in their social groups and their personal hygiene habits such as washing hands, proper sneezing and coughing etiquette are not well adhered to.

There was no significant difference in the prevalence of respiratory HAdV infections between males (10.1%) and females (9.5%) and a similar trend is reported in Korea and Egypt [22]. However, in Croatia and Jordan males were reported to be more likely to acquire HAdV infection than females (for Croatia; male-to-female ratio = 3.2:1, for Jordan; male to-female ratio 1.9:1) [30, 31].

Phylogenetic analysis showed HAdV-B and HAdV-C as the only species detected within the tested population. Of the15 isolate sequences, 10 clustered with HAdV‐B and 5 with HAdV‐C. HAdV-B and HAdV-C infections have been reported to be significantly associated with younger children[32] as we observed in this study. Reports from elsewhere have shown varying prevalence between the different species. Cameroon found that HAdV‐B infections were more common and severe than HAdV‐C [33] while Egypt and Senegal reported HAdV‐C to be more predominant [5, 25]. This difference in the HAdV species dominance can be attributed to factors such as variations in geographic location, time period and sampling methodologies.

Human adenoviruses have a worldwide spread thus most individuals have been exposed and will possess anti-HAdV antibodies [28]. In the recent years, the study of adenoviruses has gained interest due to theirpotential use in gene therapy and as delivery vectors in cancer treatments and vaccines [34]. These vectors have mainly been based on replication defective HAdV species C types 2 ad 5 in preclinical and clinical models of gene therapy [34]. However, in most populations many individuals are immune to HAdV-C2 and HAdV-C5 based vectors due to previous infection exposure which has presented a major limitation in their use [35].

The most prevalent genotype in the current study was HAdV-B3which has been reported to be associated with sporadic infection in community and institutional out breaks [36]. HAdV-B3 respiratory infections have also been reported in other parts of the world and have become a global concern [12, 37]. In the United States, HAdV-B3 respiratory infection among the civilians and military population were 34.6% and 2.6% respectively [19]. Of the HAdV-C detected, HAdV-C5 was the commonest which differs from reports in Egypt by Demian et al.[5] who reported HAdV-C1 as the commonest HAdV-C genotype identified. Despite the fact that HAdV C infections are not associated with life-threatening disease in the presence of a functional immune system, latent and persistent infections have been reported [38]. Other genotypes detected in this study were HAdV-C1, HAdV-C2 and HAdV-B7out of which HAdV-B7 and HAdV-B3have been implicated in inflammation of the upper respiratory tract and pneumonia with possible severe disease consequences [39]. Human adenovirus type B3 has been indicated to have a world-wide distribution owing to continuous mutations that has led to a high degree of heterogeneity of the hypervariable regions (HVRs) of HAdV-B3 strains circulating globally [32, 36]. Analysis of the genetic distances showed a close similarity between HAdV-B3and HAdV-B7sequences. In some studies, recombination events between HAdV-B3 and HAdV-B7 have been reported [40] which could explain the close similarity observed in this study. More discriminatory genotyping methods such as whole genome sequencing or PCR targeting different genes encoding the fiber protein, the penton base protein and the hexon protein genes could have been able to discriminate these genotypes more. In this study, we used PCR with primers that targeted the conserved loop 4 of the Hexon protein gene which has been used in other HAdV genotyping studies [18]. This could thus explain the close similarity between some of these genotypes. All the detected genotypes are commonly seen in acute respiratory infections and have been isolated from lungs in children with pneumonia. HAdV-E; a species that has commonly been associated with high rates of febrile respiratory illness in US military recruits [41] was not detected in this study and indeed has been indicated to be rare even in other studies [5].

HAdV stays in circulation throughout the year without a specific seasonal pattern [4]. The peaks of HAdV infections in the current study were observed in the months of February and March, then September, October and December. In 2014, a different trend of infection was observed where by the highest peaks appeared to be in July, August, October and December. A study in Egypt by Demian et al. [5] reported most of the isolates in his study to have been obtained during a four-month period from March through June suggesting that the peak of circulation occurred during that time frame. Some studies reported peaks to occur from late part of the wet season (March to May and September to December). Studies in Kenya reported HAdVs to be associated with higher land surface temperature (≥ 40 °C) more than cooler land surface temperature and this could explain the higher peaks in these years [4]. The presence of specific genotypes did not appear to correlate with any time period except for HAdV-B7 that first appeared in 2014 and continued appearing years afterwards. We were unable to give a conclusive seasonal, age, sex and region circulation trend of the different genotypes as not all the positives were sequenced.

## Conclusion

We confirmed the presence of different species of HAdV (HAdV-B3, HAdV-B7, HAdV-C1, HAdV-C2, HAdV-C5, HAdV-C6) associated with respiratory infections especially in children in Uganda. More studies are needed to describe the molecular dynamics of HAdV in Ugandan population through whole genome sequencing for more in-depth characterisation especially in lieu of the common adenovirus-vectored vaccines and therapuetics.

## Data Availability

Sequences analyzed in this work can be found in the GeneBank under accession numbers and links: OP784948: https://www.ncbi.nlm.nih.gov/nuccore/OP784948. OP784949: https://www.ncbi.nlm.nih.gov/nuccore/OP784949. OP784950: https://www.ncbi.nlm.nih.gov/nuccore/OP784950. OP784951: https://www.ncbi.nlm.nih.gov/nuccore/OP784951. OP784952: https://www.ncbi.nlm.nih.gov/nuccore/OP784952. OP784953: https://www.ncbi.nlm.nih.gov/nuccore/OP784953. OP784954: https://www.ncbi.nlm.nih.gov/nuccore/OP784954. OP784955: https://www.ncbi.nlm.nih.gov/nuccore/OP784955. OP784956: https://www.ncbi.nlm.nih.gov/nuccore/OP784956. OP784957: https://www.ncbi.nlm.nih.gov/nuccore/OP784957. OP784958: https://www.ncbi.nlm.nih.gov/nuccore/OP784958. OP784959: https://www.ncbi.nlm.nih.gov/nuccore/OP784959. OP784960: https://www.ncbi.nlm.nih.gov/nuccore/OP784960. OP784961: https://www.ncbi.nlm.nih.gov/nuccore/OP784961. OP784962: https://www.ncbi.nlm.nih.gov/nuccore/OP784962.

## Abbreviations

Ad: Adeno
AdV: Adenovirus
DNA: Deoxyribonucleic acid
GEIS: Global Emerging Infections Surveillance
HAdV: Human Adenoviruses
HIV: Human Immunodeficiency Virus
ILI: Influenza like illness
LARIs: Lower Acute Respiratory Infections
ml: Millilitre
MNRH: Mulago National Referral Hospital
PCR: Polymerase chain reaction
RTI: Respiratory Tract Infection
RT-PCR: Reverse transcriptase- polymerase chain reaction
WGS: Whole genome sequencing
UV: Ultra violet
℃: Degrees Celsius
